# Fostering Lifelong Learning: Integrating a Portfolio Coaching Program into an Undergraduate Medical Education (UME) Competency-Based Curriculum

**DOI:** 10.1007/s40670-025-02427-3

**Published:** 2025-06-02

**Authors:** Calvin L. Gruss, Katherine J. Walsh, William B. Cutrer, Amy Fleming, Kendra Parekh

**Affiliations:** 1 Department of Anesthesiology, Vanderbilt University Medical Center, Vanderbilt University School of Medicine, 1301 Medical Center Drive, Suite 4648, Nashville, TN 37232 USA; 2 Department of Medicine, Vanderbilt University Medical Center, Vanderbilt University School of Medicine, Nashville, TN 37232 USA; 3 Department of Pediatrics, Division of Critical Care Medicine, Vanderbilt University School of Medicine, Nashville, TN 37232 USA; 4 Department of Pediatrics, Vanderbilt University School of Medicine, Nashville, TN 37232 USA; 5 Department of Emergency Medicine, Vanderbilt University School of Medicine, Nashville, TN 37232 USA

**Keywords:** Coaching, Competency-based medical education, Curriculum development, Master adaptive learner (MAL), Medical student portfolio

## Abstract

Vanderbilt University School of Medicine (VUSM) aims to catalyze the advancement of impactful discovery, servant leadership, and lifelong learning. During a curriculum revision, VUSM fostered lifelong learning by intentionally integrating a portfolio coaching program into a competency-based curriculum. At matriculation, each student is paired with a faculty coach, and the dyads meet at regularly scheduled intervals until graduation. The program is designed to inspire and support students in reaching their full potential, assist students in creating and meeting academic goals, and support informed self-assessment to facilitate self-regulation and lifelong learning. With over 10 years of continuous refinement and extensive student participation data, this stands as one of the most mature and comprehensive undergraduate medical education coaching programs. This manuscript describes the implementation, institutional experience, and outcomes of the VUSM Portfolio Coaching Program.

## Introduction

Seeking to advance impactful discovery, servant leadership, and lifelong learning, in 2013, Vanderbilt University School of Medicine (VUSM) created Curriculum 2.0 (C2.0), an innovative competency-based curriculum [1]. A fundamental aspect of C2.0 is its longitudinal Portfolio Coaching Program, which employs an educational portfolio, data-informed self-assessment, and coach-supported, iterative development of learning goals. In undergraduate medical education, educational portfolios are known to offer various advantages, including facilitating a structured and centralized way to store and access assessment information, enhancing feedback mechanisms, providing structure for career advising, and advancing education quality improvement initiatives. Additionally, the involvement of a portfolio coach enhances the student learning experience through facilitated self-assessment, collaborative interpretation of feedback, and co-creation of practical learning goals and plans [2].

**Fig. 1:**
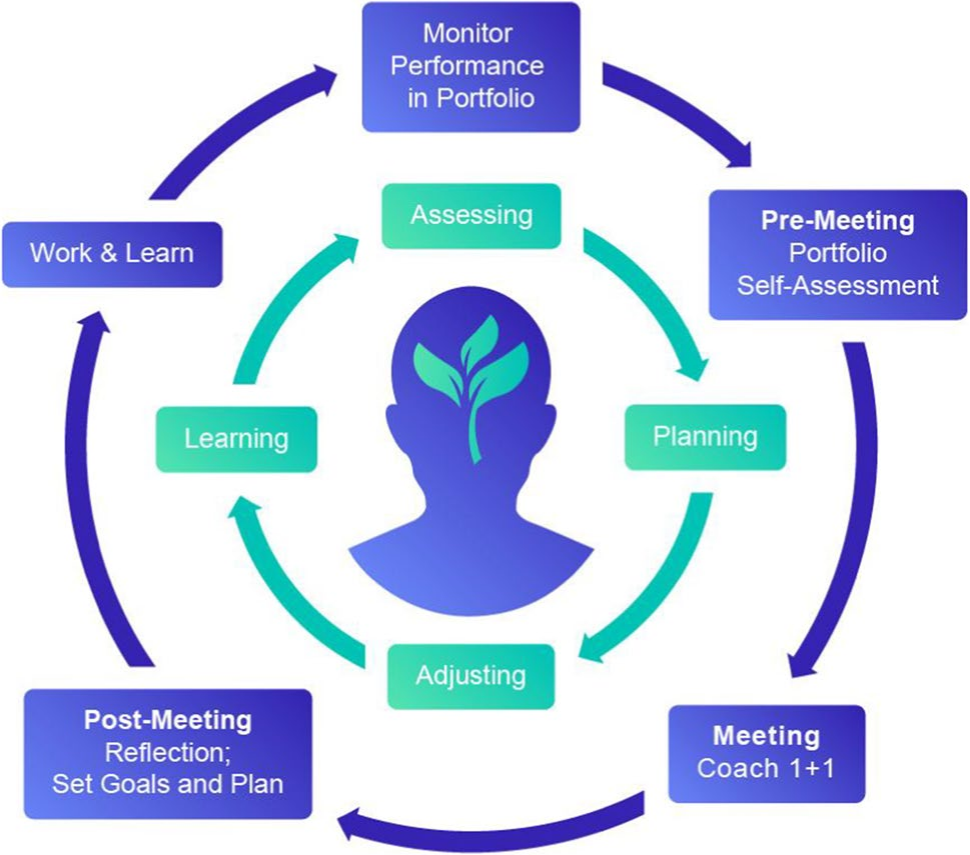
Coaching process fosters self-regulation and lifelong learning

Given C2.0’s emphasis on competency-based assessment, VUSM recognized the importance of ensuring that students had appropriate support and guidance for competency development. To meet this need, the school created the Portfolio Coaching Program, introducing a cyclical process of informed self-assessment. The program allows students to collaborate with coaches to identify their strengths and areas for growth and to formulate corresponding action plans across all competency domains, including medical knowledge, patient care, interpersonal and communication skills, practice-based learning improvement, systems-based practice, and professionalism [3]. Such analysis helps students reflect on their past performance and set goals for the future, by applying the master adaptive learner framework [4]. Briefly, the master adaptive learner (MAL) framework outlines a process by which learners can systematically approach uncertainty and change in clinical practice through self-regulated learning. It involves recognizing when new learning is needed (planning), engaging with educational resources (learning), assessing the impact of these new strategies (assessing), and then adapting one’s approach accordingly (adjusting). This cyclical process supports the continual adaptation and application of knowledge and skills in complex and dynamic clinical environments (Fig. 1).

The VUSM coaching program uses the master adaptive learner framework phases of planning, learning, assessing, and adjusting to guide students as expert learners in a process that facilitates data-driven, faculty-supported self-assessment through regular monitoring, self-assessment, coach meetings, reflection, and learning goal creation.

Unlike traditional mentoring programs, the VUSM Portfolio Coaching Program incorporates data-driven self-assessment tools and coaching interventions tailored to each student’s progress and medical education journey. Here, we share our insights and the fruitful integration of this program into the updated Curriculum 2.0.

## Approach

### I. Setting

C2.0 has three distinct curricular phases: phase 1, Foundations of Medical Knowledge, a 1-year pre-clerkship phase; phase 2, Foundations of Clinical Care, a 1-year clerkship phase; and phase 3, Immersion Phase, a 2-year post-clerkship phase. In addition, three longitudinal courses run throughout each phase: Foundations of Healthcare Delivery, Learning Communities, and Research [5, 6]. Specific competencies span six domains: medical knowledge, patient care, interpersonal and communication skills, practice-based learning and improvement, systems-based practice, and professionalism. Milestones across all courses and phases of the curriculum make it possible to track each student’s progress over time.

### II. Portfolio Coaching Program Development

VUSM’s Portfolio Coaching Program received financial support from the American Medical Association (AMA) as part of the Accelerating Change in Medical Education Initiative in 2016. Students are assigned a coach at matriculation and ideally maintain the same coach until their medical school graduation. The coaching program provides a structured environment that fosters the professional growth of students over time. Coaches play a pivotal role, promoting the practices of regular self-assessment and goal setting, and nurturing the habits of ongoing learning that parallels the MAL framework (Fig. 1). The goals of the Portfolio Coaching Program are to inspire and support students in reaching their full potential, assist students in creating and meeting academic goals, and support informed self-assessment to facilitate self-regulation and lifelong learning. Phase 1 introduces foundational self-assessment skills; phase 2 emphasizes integrating feedback from clinical experiences; phase 3 encourages advanced self-reflection for career readiness.

The rationale behind the coaching approach is intentionally preventive, meaning it proactively equips all students with the skills and strategies needed to navigate common challenges before they escalate into significant barriers. By focusing on the early development of self-regulation and reflective practice, the program helps learners identify areas of concern—such as academic difficulties, time-management struggles, or emotional stressors—and address them promptly with the support of their coaches. Self-regulation skills are acknowledged as vital components of mental health, academic success, and clinical performance [7]. Teaching students self-regulation is essential for their academic success and emotional well-being. This process involves helping students to develop the skills necessary to set their own goals, monitor their progress, adjust strategies based on feedback, and manage impulses and emotions that might influence their achievements. By learning self-regulation, students become better equipped to adapt to changing circumstances, resist distractions and temptations, and persist through challenges [8, 9]. The role of an educator in this context is to guide students through conscious and unconscious processes that enable them to take control of their actions and decisions, ensuring they align with their personal values and long-term objectives. This educational approach fosters a sense of autonomy and responsibility, preparing students for lifelong learning and personal development [10].

**Fig. 2:**
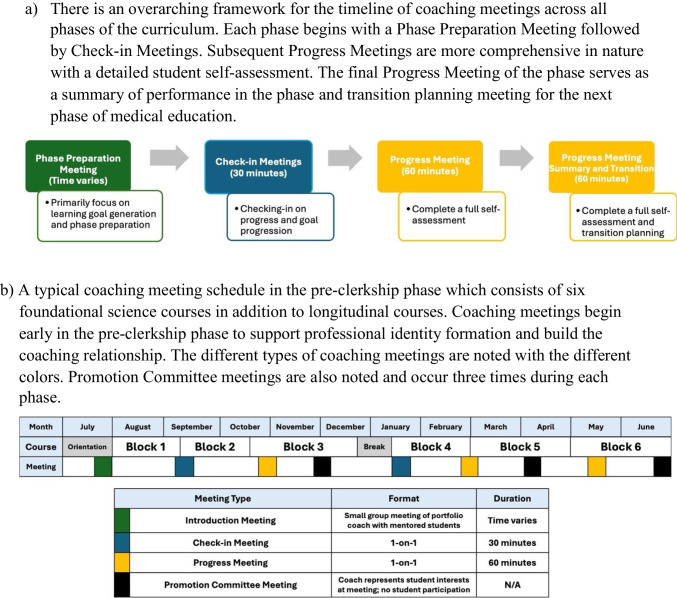
Coaching schedule

To this end, each academic year, coaches meet individually with their students approximately five times annually at regular intervals. The meeting framework is consistent across phases; however, the content of meetings varies based on the curricular phase. Typically, the first meeting of the academic year is a *Phase Preparation Meeting*, where the primary focus is on generating learning goals and preparing for that specific phase. Subsequent meetings are either *Check-in Meetings* or *Progress Meetings*. *Check-in Meetings* are scheduled for 30 min and are designed as a relationship-building touchpoint. *Progress Meetings* are scheduled for 60 min and are designed to review a student’s progress through a student self-assessment, competency review, and learning goal review (Fig. 2).

#### Role of Students

Students are expected to reflect continuously on their learning goals and competency development using assessment data provided in their electronic portfolios and their lived experiences. To promote learner agency and autonomy, prior to each coaching meeting, students identify topics to discuss with their coach and set the agenda for the meetings. For *Progress Meetings*, students will complete a pre-meeting, competency-based self-assessment with reflection. To complete the self-assessment, students receive an automated email with a link to their self-assessment. The self-assessment is completed online through an institution-developed platform. This self-assessment is then saved to the student’s portfolio and reviewed with the coach during the meeting. At the completion of coaching meetings, students update and revise their learning goals (Fig. 1). Students have a mandatory workshop during their first year on writing learning goals, with a focus on writing goals that are SMART (specific, measurable, actionable, realistic, and timebound) [11]. Students track their learning goals and plans in their electronic portfolio.

#### Role of Coaches

**Fig. 3:**
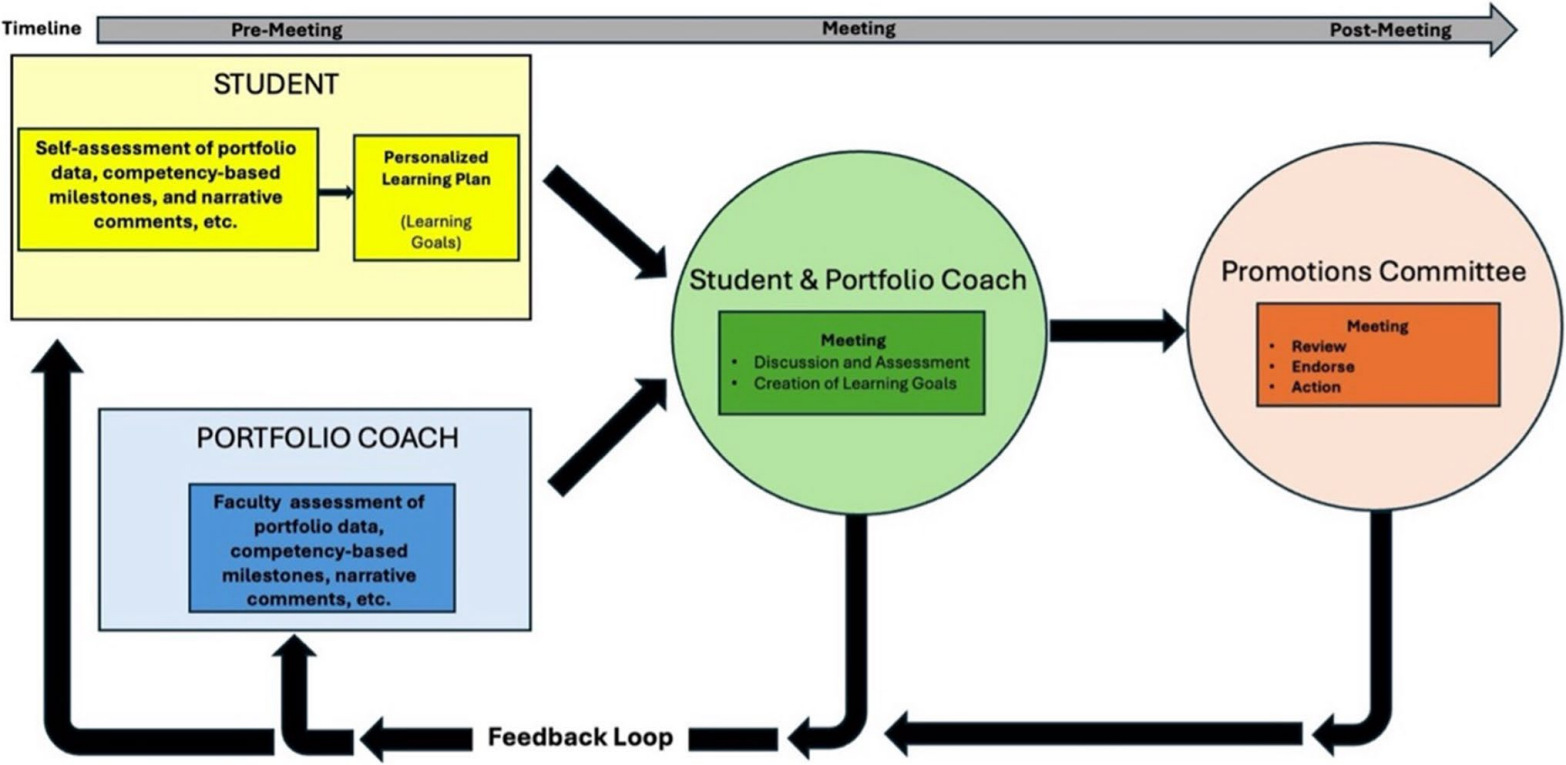
The coaching process involves independent assessment of performance data in the portfolio by the student and coach. Following independent evaluation, the student and coach meet to discuss the data together. Following discussion, the student and coach summarize their evaluation of the data. This summary is available in the student’s portfolio and can be reviewed by the promotion committee as needed

Coaches have a multifaceted role in fostering the academic and professional development of students. They are instrumental in helping students establish and achieve their academic learning goals. Drawing on self-determination theory, coaches acknowledge students’ autonomy and use the coaching relationship to help the student develop skills and competency. Coaches have full access to students’ electronic portfolios. This provides a comprehensive view of student performance data and allows the coach to ask probing questions and discern patterns in data across courses and educational experiences. Although coaches provide a written summary of student performance, they do not directly assess students, and coaching is distinct from the formal decision-making processes regarding student progression and promotion. This separation is vital in maintaining the integrity of the coaching relationship (Fig. 3).

The coaching habits fostered by the program are centered around being fully present and engaging in open-ended questioning without any preconceived agenda, judgment, or expectation. Coaches are trained to listen carefully to students, with attention to words and emotions. Reflecting on what has been said—and what is meant by it—is a key aspect of their role. The relationship between coach and student is designed to be longitudinal and built on mutual respect and trust, with coaches being approachable and active listeners. Coaches are encouraged to promote self-reflection, resisting the urge to provide immediate solutions. This helps learners develop an internal sense of control and mastery over their skills, thereby nurturing intrinsic motivation. Coaches are committed to offering high-quality, growth-oriented feedback and aid in the creation of learning goals. The coach development program used multi-modal instructional strategies to develop coaches and is described more fully in a later section.

In summary, the role of a coach encompasses guiding students through goal setting, providing evaluative feedback, encouraging independent self-reflection, and fostering a supportive and growth-minded environment. A set of coaching competencies adopted from the AMA’s Coaching in Medical Education Faculty Handbook serves as a framework for the program, holistically ensuring that students receive the best possible support in their journey through medical education [10].

### III. Program Administration and Technology

The director of the Portfolio Coaching Program reports to the senior associate dean for undergraduate medical education and oversees all aspects of the coaching program. A program manager also supports the coaching program as part of their role in curriculum management and administration.

Coaches are selected through a competitive application process and receive salary support. Each is assigned 10 to 12 students, to allow personalized attention to their cohort. An initial call for coaches is posted on standard VUSM and Vanderbilt University Medical Center list serves. Applicants are required to submit a statement of interest, a CV, and a letter of support from their chair or division director. Applications are screened by the director and the program manager, and selected candidates are invited to interview. The interview is a group interview with the director, program manager, and a student representative from the student curriculum committee. All interviewers have participated in microaggression training. Interviewers are assigned behaviorally based questions related to the goals of the coaching program. Interviewers use a standardized rubric to score the interview, and the interview score is used in conjunction with the application to make final selection decisions. Coaches are hired for a 4-year term with the option to renew.

Additionally, the program offers alternate coach positions. Alternate coaches attend coach development sessions but do not receive salary support or students to coach. Typically, they are promoted into the coach role the following academic year, after they have had the opportunity to observe the program in action and assimilate its core values and methodologies. Alternate coaches may be promoted to coach earlier to fill unexpected departures (e.g., role change, grant funding changes for an existing coach).

**Fig. 4:**
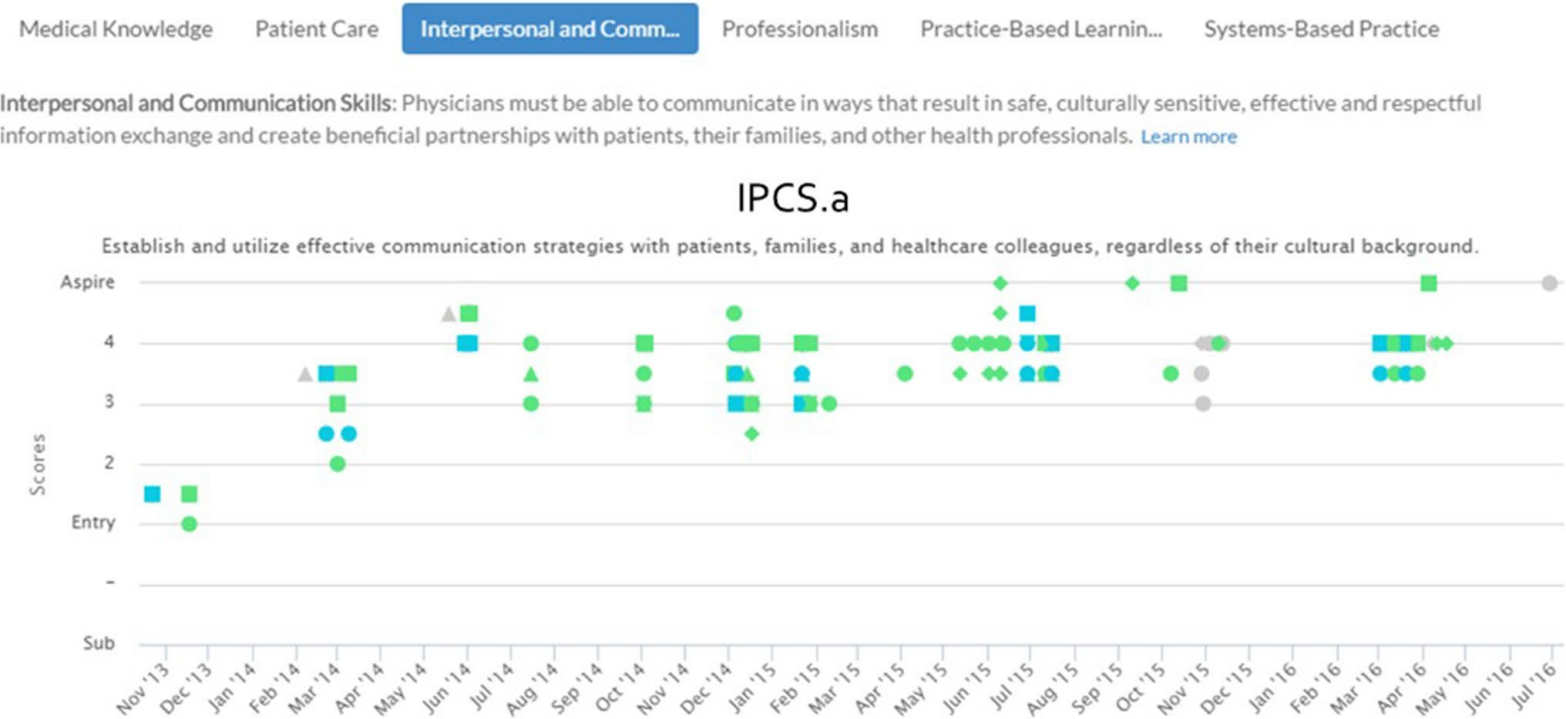
The electronic portfolio dashboard

In addition to the coaches and administrative personnel, the coaching program is supported by the Education Design and Informatics (EDI) team. Led by the associate dean for Education Design and Informatics, this team provides computer system maintenance and upgrades as well as user support. Importantly, the EDI team developed the electronic portfolio (e-portfolio) that is central to the program for both coaches and students [12, 13]. Through desktop and mobile applications, the e-portfolio monitors learner development across all years of medical school using the VUSM-specific competencies as well as the Association of American Medical College’s (AAMC) Core Entrustable Professional Activities (EPAs) for Entering Residency [14]. It contains all elements of student assessment, including course grades and scores, narrative comments, workplace-based feedback, peer feedback, self-assessments, reflections, and learning goals with learning plans. The e-portfolio system employs encryption standards compliant with FERPA guidelines and integrates assessment data via real-time synchronization with faculty evaluation tools. Vital to the coaching process is the ability to visualize aggregated competency data. To this end, the e-portfolio has competency dashboards, grouped by domain, to illustrate student progress in each area over time. Each domain dashboard has dynamic filtering capabilities, making it possible to select specific time periods or assessor groups (e.g., peer assessments or faculty assessments). Because the data in the dashboards is dynamic, clicking on any individual data point leads the user to the completed assessment (Fig. 4).

The electronic portfolio dashboard captures all elements of student assessment (course grades and scores, narrative comments, workplace-based feedback, peer feedback, student self-assessments, and learning goals), allowing students to view assessment trends over time, helping them identify strengths, weaknesses, and progress across competency domains.

The learning management system includes a scheduling tool that allows coaches to provide students with their availability. Once students select an available time slot, the system sends calendar invites to both the coach and the student. It also maintains a log of all scheduled meetings and notes if meetings were cancelled, changed, or missed. An administrative portal allows the program manager to track student completion of required self-assessments and coach completion of required forms.

### IV. Coach Development

The development of coaches is a rigorous and dynamic process informed by program evaluation data, student feedback, and coach feedback. For new coaches, a series of meetings focus on the basics of coaching and the VUSM curriculum. For all coaches, there are 1-h monthly meetings from August to May focusing on the development of coaching skills (Table 1). Continuing medical education (CME) credit is offered for the meetings. All meetings were held in person prior to the COVID-19 pandemic, pivoting to virtual during the pandemic. Since academic year 2022–2023, the new coach onboarding is held in person, and the monthly meetings are primarily virtual, with two to three in-person sessions yearly. Virtual meetings have improved attendance for coaches, allowing off-site coaches to participate more easily and making it easier for clinicians to join on time at the end of a clinic day.

**Table 1:** Example coach development calendar for an academic year

Timing	Session title/presenter	Session objectives
July	Welcome to Portfolio Coaching*	• Differentiate coaching from mentoring and advising • Describe the VUSM coaching program • Facilitate a small group session with students on coaching
July	Relational and Coaching Skills*	• Describe the coaching mindset and core habits • Construct “what” and “how” questions • Discuss common coaching tools • Conduct a brief coaching session with peers
July	Curriculum and Promotions*	• Discuss how coaching supports the school’s mission • Describe the medical education program objectives • Discuss the MD program curriculum and general assessment strategies including milestones and entrustable professional activities • Describe the promotion process and the coach role
August	Welcome to New Academic Year: Discussion and Celebration	• Recall general coaching reminders • Discuss technology and curriculum updates for the new academic year • Review coach-student meeting schedule for the new academic year • Review coach development schedule for the new academic year
August	Preparing for the 1st Meeting*	• Access the coach forms • Discuss performance expectations of students • Use the e-portfolio to review student assessment data and make determinations on performance
September	Progress and Promotions	• Discuss the promotions process and procedures • Describe how to support students of concern • Access resources to discuss progress and promotions with students
October	Time Management	• Discuss personal time management practices • Discuss best practices for medical student time management • Discuss how to support students struggling with time management and prioritization
November	Coaching Values	• Describe “what” are values, “why” we use values in coaching, “how” to discover and name values • Review specific methods to coach values, how students can use values, and potential pitfalls
December	Mentor Meet and Greet	• Meet college mentors • Compare and contrast the roles of coach and college mentor • Brainstorm how to work cooperatively to support students and common challenges
January	Medical Innovators Development Program (MIDP)** and Medical Scientist Training Program (MSTP)	• Review graduation and curriculum requirements for MIDP and MSTP students in comparison to traditional MD students • Discuss common challenges for MIDP students • Discuss common challenges for MSTP
February	Post-Clerkship Course Scheduling and Elective Counseling	• Discuss post-clerkship phase course requirements and graduation requirements • Access resources for elective counseling and planning the post-clerkship phase • Discuss best scheduling practices and common pitfalls
March	Medical Student Affairs Updates	• Discuss updates on NBME exams, USMLE Step exams, Residency Match
April	Imposter Syndrome	• Define and describe imposter syndrome • Discuss strategies to mitigate imposter syndrome and support students experiencing imposter syndrome
May	Year in Review	• Reflect on coaching experiences for past year and rapid cycle feedback from students • Plan for next academic year

*Session is primarily designed for new coaches, although all coaches are invited to attend as a refresher

**The MIDP program is a physician-engineer training track focused on interdisciplinary innovation and leadership in healthcare

Each monthly meeting includes a general overview of the academic year, including upcoming curricular and coaching events, as well as program announcements. The remainder of the session is spent on a featured topic, with topic selection curated to reflect coaches’ expressed needs and the program leaders’ strategic objectives. A sample schedule is found in Table 1. The coach development program emphasizes relational skills to prepare coaches for student meetings, with annual reviews to help ensure continuous improvement and adaptability in coaching practices. Led by experienced educators (e.g., senior associate deans) and professionals (e.g., national coaching experts), these monthly sessions provide timely and relevant support to new and returning coaches.

## Outcomes

### I. Program Evaluation

There are multiple avenues for feedback on the coaching program and its components. The program engages in continuous quality improvement as part of broader program evaluation efforts. The student curriculum committee meets monthly and provides rapid cycle feedback on the Portfolio Coaching Program. Rapid cycle feedback is a structured, iterative approach that provides timely, actionable insights, ensuring quick adaptations and continuous improvements to the Portfolio Coaching Program. There are also designated liaisons from the student curriculum committee to the Portfolio Coaching Program to help provide feedback and partner on any changes. Students complete mid-year evaluations of the program, which assess actionable items by phase, as well as end-of-year evaluations.

Students are required to participate in these annual, online evaluations, ensuring that a wide range of perspectives are captured and a near 100% response rate. Students evaluate the program’s effectiveness on a 5-point scale from “Strongly Disagree” to “Strongly Agree” across various goals, including inspiration, support in goal setting, and assistance in self-regulation. Additionally, the program’s components—such as self-assessment activities, meetings, personal learning goal creation, and the electronic portfolio tool—are rated from “Not Effective at All” to “Extremely Effective” to gauge their impact on students’ professional growth. Students are also asked to reflect on their overall experience with the program over the past year, identifying the most and least valuable elements of the program and discussing how these elements have contributed to or detracted from their professional development. Descriptive statistics (mean and standard deviation) are calculated in Excel, and comments are coded thematically to identify strengths, critical concerns, and/or areas for growth. The feedback from these evaluations, coupled with rapid cycle feedback data, forms the backbone of the program’s commitment to continuous improvement. For example, when the evaluation data indicated that coaches were not knowledgeable about the curriculum, coach development sessions discussing the various components of the curriculum were implemented and are now an annual component of the coach development program.

Student comments illustrate the program’s impact, with many highlighting the value of self-reflection opportunities facilitated by the coaching structure. Students appreciate the program’s role in helping them to understand and utilize feedback, set concrete goals, and maintain accountability—especially in preparation for residency applications. Furthermore, students recognize the benefits of the program in fostering a culture of self-improvement and humility. According to student comments, portfolio coaches provide both academic and personal support. They help connect students with mentors, offer study tips, and remain a reliable source of advice and encouragement. These actions demonstrate the coaches’ deep commitment to the students’ success.

### II. Coach Evaluation

Students complete an annual evaluation of their coach. The sustained level of student satisfaction, as documented in the analysis, reflects the program’s success in meeting its educational objectives (Table 2). Historically, students have found their coaching relationships valuable; over the years from 2018 to 2024, student feedback demonstrates continued improvement across several dimensions. The highest ratings in 2023–2024 indicate that almost all students agree or strongly agree that their coaches ask for their thoughts and opinions (4.76) and are well-prepared for meetings (4.71), invested in their success (4.69), and comfortable discussing weaknesses with them (4.63). Other notable areas include coaches helping with critical appraisal of performance data (4.59), providing a holistic view of performance (4.59), and translating assessment data into action plans (4.53). Overall, students report a positive impact on their educational experience, with a rating of 4.55 in 2023–2024.

**Table 2:** Six years of student evaluation of portfolio coaches

Statement (1 = strongly disagree, 5 = strongly agree)	Mean (standard deviation)					
	2018–2019	2019–20	2020–21	2021–22	2022–23	2023–24
My coach is adequately prepared for our meetings.	4.62 (0.4)	4.64 (0.6)	4.64 (0.6)	4.64 (0.7)	4.72 (0.5)	4.71 (0.5)
My coach is invested in my success.	4.57 (0.4)	4.65 (0.6)	4.64 (0.6)	4.62 (0.7)	4.72 (0.6)	4.69 (0.6)
I feel comfortable speaking with my coach about areas of weakness.	4.46 (0.5)	4.55 (0.8)	4.53 (0.7)	4.55 (0.8)	4.68 (0.7)	4.63 (0.7)
My coach helps me critically appraise data about my performance.	4.39 (0.6)	4.47 (0.8)	4.49 (0.7)	4.50 (0.7)	4.62 (0.7)	4.56 (0.7)
My coach helps me translate my assessment data into action plans.	4.38 (0.6)	4.44 (0.8)	4.49 (0.7)	4.51 (0.7)	4.55 (0.7)	4.53 (0.8)
Wherever my performance is at the moment, my coach helps me figure out how to move on to the next level.	4.31 (0.6)	4.37 (0.8)	4.47 (0.7)	4.45 (0.8)	4.58 (0.7)	4.51 (0.8)
My coach synthesizes my assessment data to take a holistic view of my performance.	4.46 (0.6)	4.51 (0.8)	4.55 (0.7)	4.57 (0.6)	4.62 (0.64)	4.59 (0.7)
My coach is knowledgeable about the curriculum.	4.34 (0.6)	4.40 (0.8)	4.41 (0.7)	4.45 (0.8)	4.56 (0.7)	4.43 (0.9)
My coach is aware of the personal goals I have outside of my academic performance.	4.37 (0.6)	4.36 (0.8)	4.42 (0.9)	4.47 (0.8)	4.54 (0.8)	4.51 (0.8)
My coach points me toward resources that might help in my career planning.	4.28 (0.6)	4.36 (0.9)	4.41 (0.8)	4.38 (0.9)	4.53 (0.8)	4.46 (0.9)
My coach monitors my academic progress and helps me make adjustments as indicated by my performance.	4.37 (0.6)	4.48 (0.7)	4.49 (0.7)	4.50 (0.7)	4.61 (0.62)	4.58 (0.7)
My coach asks for my thoughts, opinions, and feelings about my performance.	4.66 (0.4)	4.69 (0.6)	4.66 (0.6)	4.6 (0.6)	4.75 (0.51)	4.76 (0.6)
Overall, my experience with my coach has had a positive impact on my educational experience at Vanderbilt.	4.41 (0.6)	4.45 (0.8)	4.49 (0.8)	4.46 (0.8)	4.59 (0.74)	4.55 (0.8)

Students frequently highlight the coaches’ preparedness and investment in their success as pivotal factors in their professional development. The ability to comfortably discuss weaknesses with coaches reflects a trustful relationship that is essential for genuine self-improvement. Furthermore, the program’s emphasis on critical appraisal and actionable feedback ensures that students can effectively translate their assessments into concrete development plans. This comprehensive support system not only enhances individual student performance but also contributes to a culture of continuous improvement and excellence within the institution.

### III. Coach Development

Attendance at the coach development monthly meetings has demonstrated consistent engagement, with coach participation ranging from 9 to 22 (of a possible 30) individuals per session. The recorded totals show a significant increase in attendance over the years, barring the anomaly of 2020 due to COVID-19 disruptions.

Feedback from coaches regarding these sessions has been overwhelmingly positive. Many have highlighted the value of incorporating AAMC videos into the sessions, which have been instrumental in clarifying the goals and models of coaching. Coaches appreciate the regular cadence of the meetings, which fosters a sense of community and continuous dialogue among the cohort. The feedback underscores the excellent communication channels with the coaching leadership and the robust support system in place for troubleshooting and technical guidance.

New coaches have expressed their appreciation for the regular connection opportunities these sessions provide, affirming the clarity and direction they receive regarding coaching objectives. Some have suggested practical exercises, such as role-playing with specific coaching models, to further solidify understanding and application of these skills in real-world scenarios. This feedback is invaluable for the program’s ongoing refinement and underscores the active, community-driven approach to coach development.

## Discussion

The VUSM Portfolio Coaching Program has proven to be integral to the success of C2.0—and it has made it possible to improve our students’ educational experience year over year. Moreover, emerging longitudinal studies demonstrate that portfolio-based coaching contributes to professional identity formation and long-term career satisfaction [15], enhances clinical performance when paired with structured self-assessment tools [16], and is associated with reduced burnout and improved well-being among medical students [17].

Over the course of the program, for example, we have gained valuable insights regarding how best to align expectations between students and coaches. Each year presents the recurring challenge of clarifying the distinct role of the coach, especially in contrast to other faculty roles. While the presence of faculty mentors and deans is a strong point of the institution, distinguishing coaches’ unique contributions requires consistent clarification. To address this, annual sessions are held to seek students’ input in ways that help elucidate and refine the coaching role. Additionally, the program has recognized the need to balance formative and summative experiences for students. In response, the Portfolio Coaching meeting schedule has been refined to include more frequent encounters between coaches and students.

The program has evolved based on student feedback to better support relationship-building and cohesion within the coaching structure. In direct response to student requests for earlier connection and community, we introduced informal group-based meetings in the first semester. These small-group sessions help students and coaches establish rapport early on, creating a more comfortable environment and shared sense of community. Maintaining cohorts of students at the same training level for each portfolio coach has further strengthened these relationships, ensuring that coaches are attuned to the specific developmental stages and needs of their students. Additionally, the timing of meetings and the documentation burden have been adjusted based on student input. By optimizing the schedule and reducing unnecessary documentation, the program has become more efficient and less burdensome for students, allowing them to focus more on their learning and professional development. These changes underscore the program’s commitment to continuous improvement and responsiveness to student needs.

There remain ongoing challenges in offering a comprehensive coaching program. A study by Lomis et al., for example, notes that while schools valuing adaptive expertise have developed coaching programs to bolster students’ self-assessment abilities and the development of personalized learning plans, there exists a wide variation in the design of these coaching programs, lacking a universal standard [18]. It is important to acknowledge this variability among coaching programs for undergraduate medical education, as some, unlike the VUSM program, lack an evaluative component [19].

One challenge is balancing individualization of coaching with program standardization. While coaching is individualized, there needs to be program standards to ensure similarities of the coaching experiences. For example, the number of times a student meets with a coach should be similar and one coach should not be meeting weekly with a student while others meeting bimonthly. Similarly, students and coaches are paired at matriculation through graduation. Some students may benefit more from a different coach pairing; however, there is a lack of current evidence to best understand successful student-coach pairings and managing requests to change. Still, Pusic and colleagues highlight how coaching arrangements promote the development of metacognition and self-regulation by allowing students to analyze competency-based assessments with their coaches [20]. While coaches do not necessarily need to be in a student’s intended specialty to effectively coach, students have expressed a preference for specialty-specific coaches as they enter the Match period.

Lastly, it can be difficult to fully ascertain the impact of the coaching program on self-regulated learning skills until post-graduation. Further inquiry is addressing this area. Next steps in program development include the continued refinement of the electronic portfolio to improve the user interface and integrate additional curricular data streams to inform students’ learning goals and plans. Additionally, we are exploring the potential of artificial intelligence to support the coaching process and provide improved data analytics and visualizations.

Future studies should investigate the long-term impact of portfolio coaching on graduates’ professional behaviors and self-regulated learning, for example via alumni surveys, performance metrics in residency, or objective measures of continued goal-setting. Multi-institutional research would help determine which elements of the coaching model are most transferable across differing class sizes and resource settings. Although our student evaluations serve as a useful proxy for coach quality, we currently do not have formal pre- and post-training assessments to objectively measure the impact of our coach development sessions; future work will involve creating and deploying standardized evaluation tools to capture improvements in coaches’ facilitation and goal-setting skills. However, we believe that key tenets of our coaching program and approach will translate to other schools and coaching programs.

Limitations include that this is the experience of a single private institution in an urban setting with a relatively small class size (~ 100/class) and geographically proximate clinical learning environments. The coaching program was implemented concomitantly with a major curriculum revision, so resources were available for the development of the program, with institutional and administrative support. These contextual factors likely impact the development and maintenance of coaching programs and will vary from institution to institution.

## Conclusion

VUSM’s Portfolio Coaching Program, with over 10 years of continuous refinement and extensive student participation data, has demonstrated numerous successes and offers valuable insights for institutions developing coaching programs to support competency-based medical education. This program fosters self-assessment, reflective practice, and lifelong learning. Data indicates consistently high student satisfaction, with significant appreciation for the coaches’ preparation, investment in student success, and ability to facilitate critical self-appraisal. Ongoing development and support for both students and coaches underscore the program’s commitment to continuous improvement. This innovative approach prepares students to navigate the complexities of modern healthcare, emphasizing both clinical excellence and personal growth.

## Data Availability

Data are available from the corresponding author upon request.
